# Study protocol: Close Assessment and Testing for Chronic Graft-vs.-Host disease (CATCH)

**DOI:** 10.1371/journal.pone.0298026

**Published:** 2024-05-16

**Authors:** Joseph Pidala, Paul A. Carpenter, Lynn Onstad, Steven Z. Pavletic, Betty K. Hamilton, George L. Chen, Nosha Farhadfar, Marcie Hall, Stephanie J. Lee

**Affiliations:** 1 Department of Blood and Marrow Transplantation and Cellular Immunotherapy, Moffitt Cancer Center, Tampa, FL, United States of America; 2 Clinical Research Division, Fred Hutchinson Cancer Center, Seattle, WA, United States of America; 3 Department of Pediatrics, University of Washington, Seattle, WA, United States of America; 4 National Cancer Institute, Center for Cancer Research, National Institutes of Health, Bethesda, MD, United States of America; 5 Blood and Marrow Transplant Program, Taussig Cancer Institute, Cleveland Clinic, Cleveland, OH, United States of America; 6 University of Texas MD Anderson Cancer Center, Houston, TX, United States of America; 7 Division of Hematology/Oncology, University of Florida College of Medicine, Gainesville, FL, United States of America; 8 Department of Medicine, University of Washington, Seattle, WA, United States of America; PLOS ONE, UNITED KINGDOM

## Abstract

Chronic graft-versus-host disease (GVHD) is an immune-mediated disorder that causes significant late morbidity and mortality following allogeneic hematopoietic cell transplantation. The “Close Assessment and Testing for Chronic GVHD (CATCH)” study is a multi-center Chronic GVHD Consortium prospective, longitudinal cohort study designed to enroll patients before hematopoietic cell transplantation and follow them closely to capture the development of chronic GVHD and to identify clinical and biologic biomarkers of chronic GVHD onset. Data are collected pre-transplant and every two months through one-year post-transplant with chart review thereafter. Evaluations include clinician assessment of chronic GVHD and its manifestations, patient-reported outcomes, multiple biospecimens (blood, saliva, tears, buccal mucosa and fecal samples, biopsies of skin and mouth), laboratory testing, and medical record abstraction. This report describes the rationale, design, and methods of the CATCH study, and invites collaboration with other investigators to leverage this resource.

**trial registration**: This study is registered at www.clinicaltrials.gov as NCT04188912.

## Introduction

Chronic graft-versus-host disease (GVHD) is a common immune-mediated disorder following allogeneic hematopoietic cell transplantation (HCT). This complication develops in 10–30% of those who survive at least 100 days and has a median time to onset of 4–6 months after HCT [1–3]. Chronic GVHD is associated with worse quality of life [4–6], prolonged duration of immunosuppressive therapy (IST) [7, 8], and higher non-relapse mortality, but also a lower malignancy relapse rate [9]. Treatment is largely empiric although there are currently three FDA-approved treatment options: ibrutinib, belumosudil and ruxolitinib [10].

The close monitoring of patients post-HCT provides a unique opportunity to observe and characterize the development of the distinctive auto/alloimmune syndrome of chronic GVHD.

Animal models have identified inflammatory, immunologic and tissue response pathways operative in chronic GVHD, but direct applicability to humans remains unclear, especially the basis for its heterogeneous phenotypes. Previous work has been done to advance biologic understanding through infrequent and late assessment of large numbers of patients to gain sufficient power for analyses. A major limitation of previous studies was not having baseline samples before onset of systemic IST. The CATCH Study (Close Assessment and Testing for Chronic GVHD) differs because it assesses a smaller number of patients more frequently to “catch” the onset of human chronic GVHD clinical manifestations to better understand its early biology with the help of parallel collection of tears, saliva, oral and fecal microbiome, blood samples and skin and oral biopsies. Study participants are assessed before HCT to establish a baseline and provide “control” samples, and then every two months through the first year after HCT. This study design will allow participants to be their own controls as well as allowing comparison of those who do and do not develop chronic GVHD at similar timepoints after HCT. The study objective of CATCH is to test whether profile changes in blood proteins and cell subsets or bodily fluids and tissues predict chronic GVHD onset, severity and organ involvement.

This paper outlines the design and methods of the CATCH study and provides information about accessing materials and data from the study.

## Methods

### Population

Patients are eligible for this study if they are age ≥18 years, scheduled for allogeneic HCT from any donor for any indication with a risk of chronic GVHD of ≥25%, have ability and willingness to comply with the study assessment schedule, and ability to communicate in English or Spanish, to allow completion of patient surveys and study procedures. Patients with a chronic GVHD risk of ≤25% (umbilical cord blood, bone marrow graft with post-transplant cyclophosphamide, or use of anti-thymocyte globulin, alemtuzumab, or ex-vivo T-cell depletion) are excluded as they are unlikely to be informative to justify the intensive follow up assessments. Other exclusion criteria include hematologic malignancy with active disease at time of HCT (measurable residual disease is allowed), HCT-comorbidity index >4 [11], prior allogeneic HCT, prior autoimmune disease with ongoing symptoms or need for treatment, history of noncompliance, or inability to comply with study requirements (due to any geographic, logistic, social, or other factors). The protocol is approved by a central IRB, the Fred Hutchinson Cancer Center Institutional Review Board (initial approval date April 10, 2019, protocol #10134). All participants provide written informed consent. The study opened to accrual on September 13, 2019, and enrollment is ongoing. The consent form allows genetic studies and sharing of specimens and clinical data. Participating transplant centers and investigators are listed in the **S1 Appendix**.

### Study design

This is a prospective, longitudinal observational study. No therapeutic interventions are mandated by the protocol. Target enrollment is approximately 200 patients at 7 institutions prior to HCT to obtain at least 180 evaluable patients, however the final enrollment numbers will be determined by the number of participants who develop chronic GVHD requiring systemic immunosuppression.

**Fig 1:**
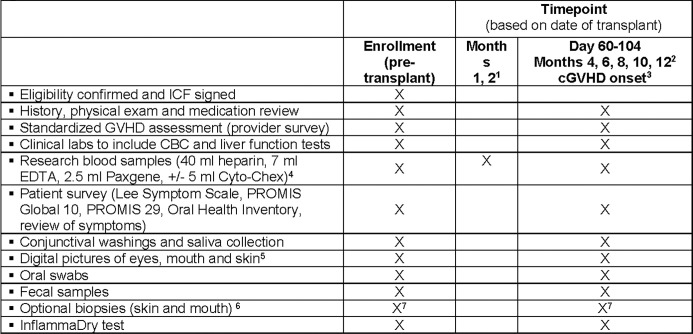
Schedule of enrollments and assessments on study. ^1^ Do not collect month 2 if a full study visit is conducted between days 60–74; acceptable window for these visits is +1–7 days. ^2^ Acceptable window for these visits is +1–30 days. To calculate target date for these visits, 1month = 30 days. ^3^ Onset visit only required if diagnosis date is more than 14 days before or after another study visit. ^4^ Cyto-Chex collected only at onset of cGVHD (per NIH consensus criteria). ^5^ Pretransplant for all subjects, and after transplant only if any abnormalities are detected. ^6^ Skin biopsy at enrollment, day 60–104, month 12, and initiation of systemic IST for chronic GVHD. Oral biopsy at day 60–104 and initiation of systemic IST for chronic GVHD.

Patients are enrolled prior to HCT. Formal study assessments occur at enrollment, day 60–104 post-HCT, and then every other month from month 4 through 12 post-HCT, as well as at time of chronic GVHD diagnosis (if this is more than 14 days from other scheduled visits). Longer-term follow up beyond these assessments is done by chart review. At each time point, patients and clinicians report information on chronic GVHD, and data and samples are collected (**Fig 1**). Additional research blood samples are collected at months 1 and 2 post-HCT. Acceptable windows for sample collection are +/- 7 days for months 1 and 2, and then +/- 30 days for subsequent time points. This study is registered at www.clinicaltrials.gov as NCT04188912.

### Data and sample collection

**Fig 1** shows the data and sample collection schedule. Clinical data pertaining to chronic GVHD onset, severity, treatment and organ involvement, relapse and death are collected via clinician-completed forms and chart review. In the setting of relapsed disease or participant withdrawal from study procedures, patients are given the option of ongoing data abstraction by medical record review only (no further study procedures). Patients who consent but do not proceed to HCT or who miss the day 60–104 visit are taken off study and replaced (no further follow up). Data are cleaned every 3 months using customized programs for range and logic checking. Queries to sites are made as necessary to reconcile discrepancies. Provider training occurred via written materials, in person meetings, web presentations, and ongoing discussions.

### Provider assessment form

The provider reports data as recommended by the NIH Consensus Conference for skin, eye, mouth, gastrointestinal, joint, genital and pulmonary chronic GVHD involvement, as well as the presence of specific clinical manifestations such as bronchiolitis obliterans syndrome. Liver and pulmonary function test results are abstracted from the chart [12, 13].

### Medical records abstraction

Information on patient, donor, and transplant characteristics, acute GVHD diagnosis, chronic GVHD presentation, and current status is collected from institutional databases or chart review. Immunosuppressive medications at the time of study visits and any given between visits are captured. Endpoint assessments (relapse, death, resolution of chronic GVHD, or discontinuation of immunosuppression) are recorded. Clinical lab values, biopsy results, and pulmonary function test results are captured.

### Research blood samples

49.5 mL peripheral blood is collected and transported directly to the processing laboratory [14]. 47 mL is collected in anticoagulant tubes (40 ml in heparin and 7 ml in EDTA) and separated as soon as possible into plasma and peripheral blood mononuclear cells (PBMC) using Ficoll. 2.5 mL of whole blood is placed in a PAXgene tube for later RNA isolation. If a patient develops chronic GVHD, an additional one-time 5 mL of blood is also collected in a Cyto-Chex tube for flow cytometry.

### Patient survey

Participants complete the following patient-reported outcome surveys: Lee Symptom Scale (30 items, 2 minutes) [15–17], PROMIS (Patient-Reported Outcomes Measurement Information System [18])-Global 10 (10 items, 1 minute), PROMIS 29 (30 items, 3–5 minutes) and the Oral Health Inventory for oral health-related QOL (14 items, 1 minute) [19]. They are also asked a standardized review of systems at each study visit using eight core items (1 minute) of the Patient-Reported Outcomes Version of the Common Terminology Criteria for Adverse Events (PRO-CTCAE) that are not covered in the other instruments [20, 21]. Total time to complete the battery is approximately 10 minutes.

### Conjunctival washings, MMP-9 testing, and saliva collection

Tear fluid is collected by instilling approximately 50 μL preservative-free Refresh Optive artificial tears to each eye, having the subject abduct, adduct, elevate and depress the eye to rinse the ocular survace, and collecting samples 15 seconds later via microcapillary tubes, anticipating 30–35 μL recovery. Matrix metalloproteinase 9 (MMP-9) testing (InflammaDry) is performed on both eyes. Validity of the MMP-9 testing was assured with built in positive controls. Unstimulated saliva is collected into a tube over 5 minutes and immediately placed on ice for transport directly to the processing laboratory. The amount of saliva collected is recorded.

### Digital pictures of eyes, mouth and skin

Digital pictures of the eyes, mouth and skin are performed pre-transplant and if any abnormalities are detected after transplant (instructions provided in manual of operations).

### Oral swabs

Swabs of the oral mucosa for microbiome analysis are taken at 2 locations: dorsal tongue and buccal mucosa.

### Fecal samples

Fecal samples are collected at home and transported at room temperature, then frozen at -80 degrees on day of receipt. Samples are stored for batch microbiome analysis.

### Biopsies (skin and mouth)

Punch biopsies of the skin and oral mucosa are performed before HCT (skin only), at day 100 (both), one year (skin only) and if chronic GVHD develops (both), according to safety parameters at each site. Oral biopsies were performed on an affected area for those with chronic GVHD. Skin and oral biopsies are optional for the participant. Biopsies are stored in two ways: frozen in optimal cutting temperature (OCT) media (until December 2022) or Cryostor (after December 2022), and formalin-fixed, paraffin-embedded.

### Biostatistical considerations

This is a longitudinal observational study with one baseline assessment and serial follow-up assessments for each participant. The target enrollment is 180 evaluable patients. Enrollment will be extended if fewer than 55 evaluable patients develop chronic GVHD. Analyses are primarily descriptive. The levels, proportions, and trajectories of cytokines, chemokines, proteins and cellular populations will be analyzed and compared between patients who develop chronic GVHD and those who do not, with attention to chronic GVHD organ manifestations and symptoms. The clinical utility of these chronic GVHD prognostic biomarkers will be evaluated. Analysis of study data will describe missing data, examine mechanisms of missingness, and attempt to account for them. These methods include a comparison of those with and without missing data, documentation of sources of missing data, and evaluations of the pattern of missingness.

### Collaboration with other investigators

The Chronic GVHD Consortium invites collaboration with other investigators who wish to access the data and research samples derived from this study. Before data or samples can be provided, a concept sheet must be discussed by Consortium members and approved by the Consortium Principal Investigator. All ancillary studies require IRB approval or waiver at both the center(s) providing data/samples and the receiving institution(s), as well as a materials transfer agreement or data use agreement if a subcontract is not in place. Approval of the collaboration by the National Cancer Institute may also be required. Data and samples are de-identified, with clinical information and samples labeled with a study ID only.

## Results

Enrollment started in 2019 and continues. **Table 1** shows characteristics of the cohort so far. As of 10/1/2023, 186 evaluable participants have been enrolled among whom 56.2% (95% CI: 43.2%, 67.2%) had developed any chronic GVHD by 26 months and 29.2% (95% CI: 21.2%, 37.7%) had developed chronic GVHD requiring IST. **Table 2.**

**Table 1:** Patient characteristics.

Variable	Category	N	Count (%)
Study site	Fred Hutchinson Cancer Center	186	101 (54.3%)
	H. Lee Moffitt Cancer Center and Research Institute		39 (21.0%)
	Cleveland Clinic		22 (11.8%)
	Roswell Park Cancer Institute		16 (8.6%)
	University of Florida		4 (2.2%)
	National Cancer Institute		3 (1.6%)
	Vanderbilt		1 (0.6%)
Recipient age at HCT in years	Median: IQR:	182	61.5 (54, 67)
	18–30	182	7 (3.8%)
	31–40		14 (7.7%)
	41–50		15 (8.2%)
	51–60		46 (25.3%)
	61–70		83 (45.6%)
	> = 70		17 (9.3%)
Patient gender	Male	185	111 (60.0%)
	Female		74 (40.0%)
Diagnosis	Acute myeloid leukemia	170	74 (43.5%)
	Myelodysplastic syndrome		37 (21.8%)
	Myeloproliferative neoplasm		21 (12.4%)
	Acute lymphocytic leukemia		15 (8.8%)
	Chronic myeloid leukemia		6 (3.5%)
	Non Hodgkins Lymphoma		4 (2.4%)
	Multiple myeloma		3 (1.8%)
	Chronic lymphocytic leukemia		2 (1.2%)
	Other		8 (4.7%)
Disease stage at transplant	Early	159	83 (52.2%)
	Intermediate		61 (38.4%)
	Advanced		15 (9.4%)
Graft Type	Peripheral Blood	170	162 (95.3%)
	Bone Marrow		8 (4.7%)
Donor type	HLA-matched unrelated donor	170	103 (60.6%)
	HLA-mismatched unrelated donor		25 (14.7%)
	HLA identical sibling		20 (11.8%)
	HLA-matched other relative		12 (7.1%)
	Haploidentical related donor		8 (4.7%)
	HLA-mismatched relative		2 (1.2%)
Conditioning Intensity	Myeloablative	168	76 (45.2%)
	Reduced intensity		59 (35.1%)
	Non-myeloablative		33 (19.6%)
GVHD prophylaxis	CNI+MTX-based	161	149 (92.6%)
	Post-HCT Cy		3 (1.9%)
	Other		9 (5.6%)
Donor-recipient sex match	Female into male	173	44 (25.4%)
	Other		129 (74.6%)
Donor-recipient CMV match	Negative/negative	167	61 (36.5%)
	Negative / Positive		36 (21.6%)
	Positive/Negative		21 (12.6%)
	Positive/ Positive		49 (29.3%)
Grade II-IV acute GVHD	Yes	175	53 (30.3%)
	No		122 (69.7%)
Follow-up of survivors (months)	Median: IQR:	144	10.5 (5, 12)

**Table 2:** Study endpoints.

Endpoint	N evaluable	CI (95% CI)
Any chronic GVHD by 14 months	157	44.9% (35.2%, 54.1%)
Any chronic GVHD by 26 months	157	56.2% (43.2%, 67.2%)
Chronic GVHD requiring systemic IST by 14 months	157	29.2% (21.2%, 37.7%)
Chronic GVHD requiring systemic IST by 26 months	157	29.2% (21.2%, 37.7%)

## Summary and conclusions

The Chronic GVHD Consortium “CATCH” Study aims to improve biologic understanding of chronic GVHD development after HCT. We hope to detect the earliest clinical and biologic changes that presage chronic GVHD by collecting clinically annotated pre-transplant baseline and early post-transplant biological samples during the high-risk period for developing chronic GVHD. By prospectively collecting comprehensive data in a large population treated at many centers, with heterogeneous chronic GVHD manifestations and severity, we have a rich longitudinal database to inform these analyses. This information may be used to design pre-emptive and early intervention studies.

This study has some limitations. Enrollment continued during the COVID-19 pandemic but the speed of enrollment and completeness of data were affected. Some procedures could not be performed due to infectious disease control measures, and patients were reluctant to spend any extra time in the clinic. Although every two months is relatively frequent, we may still have missed relevant samples and symptoms that would have been informative for impending chronic GVHD. The sample size is relatively small because of the intensive follow-up procedures required.

Investigators interested in the clinical data, research forms, database structure or research samples available from this cohort study should contact the corresponding author for procedures on how to apply for access. We welcome collaboration with other investigators involved in chronic GVHD research.

## Supporting information

S1 Checklist SPIRIT 2013 checklist: Recommended items to address in a clinical trial protocol and related documents*. (DOC)

S1 Protocol Close Assessment and Testing for Chronic GVHD (The CATCH study). (DOCX)

S1 Appendix Participating sites from the Chronic GVHD Consortium. (DOCX)
