# Supplementary material for: Study protocol: Close Assessment and Testing for Chronic Graft-vs.-Host disease (CATCH)
Source: PLoS One. 2024 May 16;19(5):e0298026. doi: 10.1371/journal.pone.0298026 (PMC11098321; doi:10.1371/journal.pone.0298026)
Supplement: S1 Protocol — (DOCX) [file pone.0298026.s002.docx]

| **Close Assessment and Testing for**  **Chronic GVHD**  **(The CATCH Study)** | | |
| --- | --- | --- |
|  | | |
| Protocol History   \| Original \| Version 1.0 \| 5 March 2019 \| \| --- \| --- \| --- \| \| Modification \| Version 1.1 \| 16 Sept 2019 \| \| Modification \| Version 1.2 \| 6 Feb 2020 \| \| Modification \| Version 1.3 \| 19 Jan 2021 \| \|  \|  \|  \| | | |
| **Study Sponsor:** | National Institutes of Health | |
|  | | |
| **Principal Investigator:** | | Stephanie Lee, MD, MPH  Fred Hutchinson Cancer Research Center  1100 Fairview Ave. N., D5-290  Seattle, WA 98109  Phone: 206.667.5191  E-mail: [sjlee@fredhutch.org](mailto:sjlee@fredhutch.org) |
| **Participating Sites:** | | Fred Hutchinson Cancer Research Center, Seattle, WA  National Cancer Institute, Bethesda, MD  H. Lee Moffitt Cancer Center, Tampa, FL  Cleveland Clinic, Cleveland, OH  Roswell Park Cancer Center, Buffalo, NY  Vanderbilt University, Nashville, TN |
|  | | |

**Table of Contents**

[1. Background and Rationale 3](#_Toc484004841)

[2. Specific Aims 3](#_Toc484004842)

[3. Subject Selection 3](#_Toc484004843)

[4. Study Procedures 4](#_Toc484004844)

[5. Biostatistical Considerations 8](#_Toc484004845)

[6. Risks and Discomforts 10](#_Toc484004847)

[7. Potential Benefits 10](#_Toc484004848)

[8. Monitoring and Quality Assurance 10](#_Toc484004850)

[9. References 12](#_Toc484004851)

# 1. Background and Rationale

Allogeneic hematopoietic cell transplantation (HCT) can cure hematologic malignancies and other bone marrow and immunodeficiency diseases, but its success is limited by high treatment-related morbidity and mortality. Replacement of the host immune system with donor cells results in 20-50% of patients developing chronic graft-versus-host disease (cGVHD), a debilitating iatrogenic condition that is the leading cause of non-relapse mortality in transplant survivors otherwise cured of their malignancies.^1-5^ Affected patients suffer from organ dysfunction and treatment toxicity, causing physical, functional and psychosocial deficits, inability to return to work, and poor quality of life (QOL) compared to patients who do not develop cGVHD.^6-11^

Although new animal models have provided insights into inflammatory, immunologic and tissue response pathways leading to nonhuman cGVHD, direct applicability in the human system is still unclear. Treatment is largely empiric. The heterogeneous phenotypes are unexplained. This protocol aims to advance our biologic understanding of human cGVHD by conducting a study that allows us to “watch” human cGVHD as it develops. The CATCH Study (Close Assessment and Testing for Chronic GVHD) will enroll 200 patients at 6 institutions prior to HCT to obtain 180 evaluable patients. After extensive pre-HCT baseline assessments and infusion of donor cells, participants will be assessed frequently after HCT for eye, mouth, lung, liver and skin chronic GVHD. At these visits, biological specimens, patient-reported outcomes, spirometry, and optical coherence tomography will also be collected. This project exploits the fact that human cGVHD is an iatrogenic disease, with high incidence and predictable onset within the first year post-HCT. Previous work has tried to facilitate biologic understanding through infrequent measurement of large numbers of patients to gain sufficient power for analyses. This project differs because it focuses on biologic understanding of the early development of human cGVHD through intensive follow up of a smaller number of individuals.

Completion of this project will lead to a better understanding of human cGVHD at the tissue level.

# 2. Specific Aims

Specific Aim 1: To test the hypothesis that levels of cytokines, chemokines, other proteins and microbiome profiles found in bodily fluids and blood will presage development of clinical cGVHD.

Specific Aim 2: To test the hypothesis that the number and functionality of cellular populations, particularly monocytes and regulatory T cell populations, will predict development of clinical cGVHD.

Specific Aim 3: To test the hypothesis that tissue alterations in skin, mouth and eyes measured by biopsy and/or advanced bioimaging techniques will predict development of organ-specific cGVHD.

# 3. Subject Selection

## 3.1 Inclusion criteria:

1. Adults age 18 or older
2. Scheduled for allogeneic HCT from any donor for any indication, with a risk of cGVHD of >25% (see below in exclusion criteria for treatment plans with a cGVHD risk <25%).
3. Ability and willingness to comply with the intensive assessment schedule including evaluation every other month at a participating site.
4. Ability to communicate in English or Spanish, to allow completion of patient surveys and clear communication with the study team.

## 3.2 Exclusion criteria:

1. Receipt of umbilical cord blood, bone marrow with post-transplant cyclophosphamide (peripheral blood with post-transplant cyclophosphamide is allowed), anti-thymocyte globulin, alemtuzumab, or ex-vivo T-cell depletion. These patients are excluded because they have a cGVHD risk of <25%.
2. Hematologic malignancy with active disease at the time of transplant. Minimal residual disease is allowed.
3. Hematopoietic cell transplant co-morbidity index > 4 based on parameters known at time of enrollment.^12,13^
4. Prior allogeneic transplant.
5. Prior autoimmune disease with ongoing symptoms
6. History of noncompliance.
7. Inability to comply with study requirements due to geographic, logistic, social or any other factors.

# 4. Study Procedures

## 4.1 Study Design

We anticipate enrolling 180 evaluable patients over 3 years (approximately 60 patients per year). Patients will be enrolled prior to transplant. With 1 year of follow up, the total duration of the study is estimated to be 4 years. Patients will be seen in the clinics of the participating institutions.
 Detailed study assessments, with data and sample collection, will occur at enrollment, 60-104 days post-transplant, and then every other month starting at 4 months post-transplant, and continuing through month 12. Participants may have one additional visit, at the time that cGVHD is diagnosed, if the prior or next planned visit is more than 14 days from the diagnosis date.

## 4.2 Required Data, Assessments, and Specimens

Required elements are outlined in Table 1. The windows for data collection are broad given the observational nature of the study with an intent to adhere as close to the outlined schedule as possible with a goal of assessing patients with detailed evaluations approximately every 2 months. Study visits will take approximately 2 hours total, with 30 minutes with a physician or mid-level provider and 1 hour for testing and collection of images. Additional information is available in the CATCH Study Manual of Procedures.

**Table 1. Schedule of tests and procedures.**

|  |  | **Timepoint**  (based on date of transplant) | |
| --- | --- | --- | --- |
|  | **Enrollment (pre-transplant)** | **Months**  **1, 2^1^** | **Day 60-104**  **Months 4, 6, 8, 10, 12^2^**  **cGVHD onset^3^** |
| - Eligibility confirmed and ICF signed (4.2.1) | X |  |  |
| - History, physical exam and medication review (4.2.2) | X |  | X |
| - Standardized GVHD assessment (provider survey) | X |  | X |
| - Clinical labs to include CBC and liver function tests (4.2.3) | X |  | X |
| - Research blood samples (40 ml heparin, 7 ml EDTA, 2.5 ml Paxgene, +/- 5 ml Cyto-Chex)^4^ (4.2.4) | X | X | X |
| - Portable spirometry^5^ (4.2.5) |  |  | X |
| - Patient survey (Lee Symptom Scale, PROMIS Global 10, PROMIS 29, Oral Health Inventory, review of symptoms) (4.2.6) | X |  | X |
| - Conjunctival washings and saliva collection (4.2.7) | X |  | X |
| - Digital pictures of eyes, mouth and skin (4.2.8)^6^ | X |  | X |
| - Optical coherence tomography (4.2.9) ^8^ | X |  | X |
| - Oral swabs (4.2.10) | X |  | X |
| - Fecal samples (4.2.11) | X |  | X |
| - Biopsies (skin and mouth) (4.2.12) | X^7^ |  | X^7^ |
| - InflammaDry test | X |  | X |

^1^ Do not collect month 2 if a full study visit is conducted between days 60-74; acceptable window for these visits is +/- 7 days.

^2^ Acceptable window for these visits is +/- 30 days. To calculate target date for these visits, 1month=30 days.

^3^ Onset visit only required if diagnosis date is more than 14 days before or after another study visit

^4^ Cyto-Chex collected only at onset of cGVHD (per NIH consensus criteria).

^5^ Not collected if pulmonary function tests are available

^6^ Pretransplant for all subjects, and after transplant only if any abnormalities are detected

^7^ Skin biopsy at enrollment, day 60-104, month 12, and initiation of systemic IST for chronic GVHD. Oral biopsy at day 60-104 and initiation of systemic IST for chronic GVHD.

^8^ Optical coherence tomography collected for selected sites, patients, and time points: pre transplant and day 60-104, 6 and 12 mos, and cGVHD onset

***The following items in the schedule above are for data collection only: Clinical labs, spirometry (if done as standard-of-care), history and physical exam (if done as standard-of-care) and medication review (if done as standard-of-care).**

4.2.1 Eligibility confirmed and ICF signed

Potential participants are identified by procedures developed locally. An IRB waiver will be obtained to allow screening via chart review. Once a potentially eligible patient is identified, a member of the study team will approach the patient for consent. The background, rationale and study requirements will be described to the patient and the patient given the opportunity to ask questions. No study-specific procedures will take place until signed informed consent is obtained from the patient. Children and adults who are not able to provide informed consent are not eligible for this study.

4.2.2 History, physical exam and medication review

Physical exams will include all organ evaluations needed for the study. Standardized assessment and documentation will be collected for the skin, eye, mouth, lung, liver, upper and lower GI tract, esophagus, joints and external genitalia according to the 2014 NIH Consensus Criteria. Any other cGVHD involvement will be documented. Interim history, review of systems and medication lists will be documented.

4.2.3 Clinical labs to include CBC and liver function tests

Data from clinical labs will be collected. These labs should include at a minimum a CBC and liver function tests as per standard of care. If other pertinent values are available, they will also be collected.

4.2.4 Research blood samples

49.5 mL peripheral blood will be collected and transported directly to the processing laboratory.^23^ 47 mL will be collected in anticoagulant tubes (40 ml in heparin and 7 ml in EDTA) and separated as soon as possible into plasma and PBMC using Ficoll. 2.5 mL of whole blood will be placed in a PAXgene tube for later RNA isolation. If a patient develops cGVHD, an additional one-time 5 mL of blood will also be placed in a Cyto-Chex tube for flow cytometry. Clinicians will have discretion about limiting the amount of research blood drawn.

4.2.5 Portable spirometry

FEV1, FVC and FEF 25-75 will be measured using a portable spirometer. A decline of more than 10% in predicted FEV1 over 3 months will prompt notification of the treating clinician. Standard of care is formal PFTs pre-transplant, at 3 and 12 months post-transplant, and if cGVHD develops. This study will collect the results from full pulmonary function tests done as standard of care. If formal PFTs are performed, then portable spirometry is not necessary.

4.2.6 Patient survey

Participants will complete patient-reported outcome surveys consisting of the Lee Symptom Scale (30 items, 2 minutes), the PROMIS (Patient-Reported Outcomes Measurement Information System) global 10 (10 items, 1 minute), the PROMIS 29 (30 items, 3-5 minutes) and the Oral Health Inventory for oral health-related QOL (14 items, 1 minute).^14^ They will also be asked a standardized review of systems at each study visit using eight core items (1 minute) of the Patient-Reported Outcomes Version of the Common Terminology Criteria for Adverse Events (PRO-CTCAE) that are not covered in the other instruments.^15,16^ The total time to complete the battery is approximately 10 minutes.

4.2.7 Conjunctival washings and saliva collection

Tear fluid will be collected by instilling approximately 50 µL preservative-free Refresh Optive Sensitive artificial tears to each eye and collecting samples 1 minute later via microcapillary tubes, anticipating 30-35 µL recovery. MMP-9 testing (InflammaDry) will be performed. Unstimulated saliva will be collected into a tube over 5 minutes and immediately placed on ice for transport directly to the processing laboratory. The amount produced will be recorded.

4.2.8 Digital pictures of eyes, mouth and skin

Digital pictures of the eyes, mouth and skin will be performed pre-transplant and if any abnormalities are detected after transplant.

4.2.9 Optical coherence tomography (OCT)

OCT is a non-invasive, high-resolution imaging method that uses visible light to reconstruct three-dimensional images with micrometer resolution. Participants will be positioned on a bed and 5 second images taken of standardized skin sites (shoulders, upper inner and lower ventral forearms, right lower quadrant abdomen, bilateral thighs and anterior lateral lower extremities, as well as any areas of skin cGVHD). Photography will document the location of images and ensure consistent locations are used. An upright device allows similar images to be taken of the mouth and bilateral eyes. OCT has been used to investigate structural abnormalities in sclerosis, ^17,18^ dry eye,^19^ and dry mouth diseases.^20,21^ In Seattle, OCT will be performed in the Wang Laboratory at the University of Washington Department of Bioengineering (Fogarty Hall) or at the Seattle Cancer Care Alliance. At the NIH, OCT will be performed in the respective clinics (dermatology, ophthalmology, dental medicine). Equipment is cleaned between patients. Other sites will not be performing OCT unless they have available equipment.

4.2.10 Oral swabs

Swabs of the oral mucosa for microbiome analysis will be taken at 2 locations: dorsal tongue and buccal mucosa.

4.2.11 Fecal samples

Fecal samples will be collected at home using an established protocol and brought to each study visit and at cGVHD onset. Samples will be stored for batch analysis.

4.2.12 Biopsies (skin and mouth)

Details about the collection, handling, and storage of the biopsy samples can be found in the study lab manual. Some of the biopsies might be done for clinical purposes; those that are done solely for research are optional. Prior to skin or oral research biopsies, the available medical history will be reviewed to ensure that no medical conditions (e.g., neutropenia, thrombocytopenia, steroids >1 mg/kg/d) are contraindications to biopsies.

4.3 Study Payments and Long-Term Follow-up

## After consent, patients will be scheduled for the various sample and data collection procedures. Patients will be paid $50 for each study visit, except for those visits where optional research biopsies are obtained, for which they will be paid $100. If possible, all data will be collected from patients and clinicians at the time of clinic visits, although patients may be provided with a self-addressed, stamped envelope to return their surveys later by mail. Participants may be contacted by the study coordinator after the clinic visit to encourage them to complete and return their surveys. All data will be maintained in secured areas (locked file cabinets and password-protected electronic databases in REDCap). Clinical data (information pertaining to onset and severity of cGVHD, cGVHD treatment, relapse, and death) will be collected via chart review until the grant or study ends.

If a patient relapses with their primary disease, study data collection will cease and no additional research samples will be taken. The patient will continue to be followed by chart review, until the grant or study ends, for development of chronic GVHD and death. Patients who withdraw from the study procedures (refuse further study participation) will be asked if the study team can continue to review their medical records periodically for long-term outcomes.

Patients who sign consent but do not proceed to transplant, or who miss components of the day 60-104 visit, will be taken off study and have no further follow-up.

## 4.4 Adverse Reactions and their Management

Because of the nature of the research, adverse events related to the study are likely to be minimal and limited to complications from the biopsies or research samples, or psychosocial distress caused by questionnaire completion. Given the instruments used, it is unlikely that the questionnaires will detect any emergent psychosocial concerns. However, if a member of the study team is made aware of any such difficulties, the patient’s physician will be notified and appropriate referrals made to social or psychological services. Participants will be instructed to contact the study team immediately if they experience any adverse reaction that could be attributed to a study procedure, and they will be given appropriate after-care instructions and contact information for biopsies. Any severe adverse events believed to be related to study participation will be reported immediately to the IRB. Because this study does not involve any intervention intended to treat chronic GVHD, we will not be convening a Data Safety and Monitoring Board but will rely on the Principal Investigators and responsible IRB to monitor the study.

## Patients will be reporting their symptoms to investigators. Patients will be clearly told that they may skip over any survey question they wish and may withdraw from the study at any time. Despite our best efforts and use of validated instruments known to be well-tolerated by patients, it is conceivable that some patients may find the process upsetting.

## 4.5 Data and Specimen Sharing Plan

This study will create a biorespository of clinical data and biospecimens available for future laboratory studies. Blood, saliva, eye washings, and other samples will be processed locally and stored at the participating institutions prior to disbursement to laboratory investigators or final storage at the National Marrow Donor Program/Be The Match located in Minneapolis, MN.

Any requests for data sharing during the active funding period will be reviewed by the investigators. Criteria for release of data will include: importance of the research question in advancing knowledge; ability of already-collected or prospectively-collected data to answer the research question; adequate funding for the investigator to conduct the proposed studies; appropriate use of the materials according to the consent form signed by the participant; and demonstration of appropriate IRB approvals where the analyses will be conducted. There will also be an expectation of presentation or publication of the data regardless of the results.

All study data, including images, will be stored in a secure database using only the subject’s unique identifier. The link to the subject’s identity is maintained at the enrolling site only. If clinical or research data are released to other investigators, identifying information or images will be removed.

# 5. Biostatistical Considerations

## 5.1. Sample Size

## This is a prospective, longitudinal observational study of patients during the first year after HCT. The study will enroll 60 evaluable patients per year for 3 years to achieve 180 evaluable participants. In order to obtain 180 evaluable patients, we have inflated enrollment by 10% (total N=200) to account for patients who do not proceed to transplant, who relapse or die before day 100 or demonstrate non-compliance by missing components of the day 60-104 visit. Enrollment will be extended if fewer than 50 evaluable patients develop cGVHD, and this rate will be monitored every 6 months starting one year after enrollment commences. 90% of cGVHD cases are diagnosed within 12 months of HCT, at a median of 4-6 months after transplant.

## 5.2. Analysis plan

## The cumulative incidence of cGVHD will be calculated. The organ spectrum, severity and clustering of organs will be described. There will be 200 baseline visits for all enrolled patients (patients who do not complete the baseline visit will be replaced). Approximately 20 patients are not considered part of the evaluable cohort due to not undergoing HCT, dying or relapsing before 3 months and being excused from the cohort due to poor compliance with study requirements. Thus, 180 patients will undergo the 3 month assessment and be considered the evaluable cohort. Note that clinical visits and sample collection continue according to the same schedule after onset of cGVHD since new organ involvement can develop over time.

*5.2.1. Specific Aim 1: To test the hypothesis that levels of cytokines, chemokines, other proteins and microbiome profiles found in bodily fluids and blood will presage development of clinical cGVHD.*

This analysis will compare the levels and trajectories of proteins and microbiome populations between patients who do and do not develop cGVHD. Blood will be analyzed separately from saliva and conjunctival washings. Onset of cGVHD will be treated as a time-to-event endpoint, using Cox regression with monthly levels or slopes of the markers entered as time-dependent covariates. While the goal of this study is to understand the biologic changes that occur in patients who develop cGVHD and to compare these changes to patients who do not have cGVHD, logistic regression and ROC analysis at fixed landmark time points will also be used to characterize the potential clinical utility of the markers in predicting future cGVHD in terms of sensitivity, specificity and positive predictive value. Assuming approximate normality of an individual marker (suitably transformed) or a ‘score’ derived from a panel of markers, a difference in means of 1 standard deviation (SD) unit between those who do and do not develop cGVHD over a specified time period corresponds to an area under the ROC of approximately 0.77. This is a minimal threshold for clinical utility, though it does not guarantee useful levels of sensitivity and positive predictive value. The planned sample size should provide adequate power (80-99%) to detect such differences, depending on the numbers at risk at a landmark time and the proportion that develop cGVHD subsequently, as well as the degree of adjustment for multiple comparisons. Currently, the following proteins are of highest interest: IL-1, IL-6, IL-8, IL-10, IL-15, IL-17, IL-21, IL-27, IFNγ, TNFα, TGFβ, sBAFF, CXCL4, CXCL9, CXCL10, CCL2, and MCP-1, but the list may evolve before actual testing. Biologic profiles will be correlated with physical exam findings and patient reported symptoms. Analyses of trajectories after cGVHD onset will also be performed to understand changes that occur with treatment.

*5.2.2. Specific Aim 2: To test the hypothesis that the number and functionality of cellular populations, particularly monocytes and regulatory T cell populations, will predict development of clinical cGVHD.*

### This analysis will be similar to that described in 5.2.1 in that we will compare the levels, proportions and trajectories of different cellular populations between those with and without cGVHD, and with different cGVHD organ involvement and symptoms. The following cell subtypes are of highest interest: Th17, FOXP3^+^ T regulatory cells, FOXP3^-^ T regulatory type 1 (TR1) cells, T follicular helper cells, activated B cells, B regulatory cells, and monocytes but the list may evolve before actual testing.

*5.2.3. Specific Aim 3: To test the hypothesis that tissue alterations in skin, mouth and eyes measured by biopsy and/or advanced bioimaging techniques will predict development of organ-specific cGVHD.*

### Histologic findings, RNA expression profiles, OCT findings and digital image interpretations will be compared between patients who do and do not develop cGVHD or who have different cGVHD clinical phenotypes and symptoms.

## 5.3 Handling Missing Data

# We anticipate significant missing data due to patient illness and hospitalization or relapse but will try to minimize missing data due to patient noncompliance or logistics. In order to devote resources to the most informative cohort, some patients who complete the baseline visit will not continue with additional visits (see above). The pattern and predictors of missing data will be characterized. Most biologic analyses will be performed on available data without any attempt at imputation. Descriptive analyses will acknowledge missing data and provide sensitivity analyses to explore potential effects of response bias on population estimates.

# 6. Risks and Discomforts

The only physical risks are from the research biopsies, research blood and bodily fluid sampling, or functional testing (e.g., spirometry) but these are anticipated to be minimal. It is possible that the self-assessments could cause emotional distress, but the risk is very low given the instruments used and our prior experience where no distress was reported. If distress is detected, the Principal Investigators will have procedures in place to contact the patient and refer them to the appropriate psychosocial support staff. As frequent evaluation by the transplant center at the time of enrollment and follow-up is required and some additional evaluation procedures may be performed if abnormalities are detected, there is the potential for additional costs incurred by the participants and/or their insurance company. However, all testing in response to detected signs or symptoms would be considered part of optimal care for a person after transplantation and thus would be clinically indicated and should be covered if a person has insurance. There are no funds provided for standard clinical care of uninsured people, and each site would need to make individual decisions in these situations. NCI will cover research costs for participants on clinical studies. There are no data requested from the transplant database or provider assessments that can cause distress to clinicians or patients. There have been no breaches of privacy or confidentiality in any Chronic GVHD Consortium studies thus far. Potential participants will be informed that the alternative is not to participate in this research and that their care will not be affected if they decline.

# 7. Potential Benefits

# Individual participants are unlikely to benefit medically from participation in this protocol, although the frequent close follow-up may detect medical issues before they would otherwise have been detected under normal care. In other studies, patients have reported satisfaction with filling out self-assessment surveys and participation in research. They may benefit by knowing they are participating in a study aimed at improving our understanding of cGVHD. We primarily hope, however, to benefit future patients. For participants, the risks of the study are minimal whether measured absolutely or relative to what we hope to learn that will benefit future patients.

# 8. Monitoring and Quality Assurance

Because this is an observational study, with minimally invasive procedures for data and sample collection, we will not be convening a Data Safety and Monitoring Board but will rely on the Principal Investigator and responsible IRB to monitor the study.

Each site is responsible for obtaining local IRB approval/inter-IRB authorization, documenting patient eligibility and consent, and developing local approaches to achieve the study aims. The Coordinating Center at Fred Hutchinson Cancer Research Center will develop and maintain the master protocol, consents, enrollment databases and data collection tools (electronic databases and patient-reported methods). Central and local study staff will work together to ensure complete and accurate data collection. Data will be cleaned on a periodic, routine basis, and data compliance and completion will be reported to the Principal Investigators so that any problems can be addressed.

# 9. References

1. Bhatia S, Francisco L, Carter A, et al. Late mortality after allogeneic hematopoietic cell transplantation and functional status of long-term survivors: report from the Bone Marrow Transplant Survivor Study. *Blood*. 2007;110(10):3784-3792.

2. Socie G, Stone JV, Wingard JR, et al. Long-term survival and late deaths after allogeneic bone marrow transplantation. Late Effects Working Committee of the International Bone Marrow Transplant Registry. *N Engl J Med*. 1999;341(1):14-21.

3. Goldman JM, Majhail NS, Klein JP, et al. Relapse and late mortality in 5-year survivors of myeloablative allogeneic hematopoietic cell transplantation for chronic myeloid leukemia in first chronic phase. *J Clin Oncol*. 2010;28(11):1888-1895.

4. Martin PJ, Counts GW, Jr., Appelbaum FR, et al. Life expectancy in patients surviving more than 5 years after hematopoietic cell transplantation. *J Clin Oncol*. 2010;28(6):1011-1016.

5. Wingard JR, Majhail NS, Brazauskas R, et al. Long-term survival and late deaths after allogeneic hematopoietic cell transplantation. *J Clin Oncol*. 2011;29(16):2230-2239.

6. Pidala J, Kurland B, Chai X, et al. Patient-reported quality of life is associated with severity of chronic graft-versus-host disease as measured by NIH criteria: report on baseline data from the Chronic GVHD Consortium. *Blood*. 2011;117(17):4651-4657.

7. Pidala J, Kurland BF, Chai X, et al. Sensitivity of changes in chronic graft-versus-host disease activity to changes in patient-reported quality of life: results from the Chronic Graft-versus-Host Disease Consortium. *Haematologica*. 2011;96(10):1528-1535.

8. Baker KS, Gurney JG, Ness KK, et al. Late effects in survivors of chronic myeloid leukemia treated with hematopoietic cell transplantation: results from the Bone Marrow Transplant Survivor Study. *Blood*. 2004;104(6):1898-1906.

9. Khera N, Storer B, Flowers MED, et al. Non-malignant late effects and compromised functional status in hematopoietic cell transplantation survivors. *J Clin Oncol*. 2011;17:995-1003.

10. Sun CL, Francisco L, Kawashima T, et al. Prevalence and predictors of chronic health conditions after hematopoietic cell transplantation: a report from the Bone Marrow Transplant Survivor Study. *Blood*. 2010;116(17):3129-3139; quiz 3377.

11. Syrjala KL, Langer SL, Abrams JR, et al. Recovery and long-term function after hematopoietic cell transplantation for leukemia or lymphoma. *Jama*. 2004;291(19):2335-2343.

12. Sorror ML. How I assess comorbidities before hematopoietic cell transplantation. *Blood*. 2013;121(15):2854-2863.

13. Sorror ML, Maris MB, Storb R, et al. Hematopoietic cell transplantation (HCT)-specific comorbidity index: a new tool for risk assessment before allogeneic HCT. *Blood*. 2005;106(8):2912-2919.

14. Slade GD. Assessing change in quality of life using the Oral Health Impact Profile. *Community Dent Oral Epidemiol*. 1998;26(1):52-61.

15. Basch E, Reeve BB, Mitchell SA, et al. Development of the National Cancer Institute's patient-reported outcomes version of the common terminology criteria for adverse events (PRO-CTCAE). *J Natl Cancer Inst*. 2014;106(9).

16. Dueck AC, Mendoza TR, Mitchell SA, et al. Validity and Reliability of the US National Cancer Institute's Patient-Reported Outcomes Version of the Common Terminology Criteria for Adverse Events (PRO-CTCAE). *JAMA Oncol*. 2015;1(8):1051-1059.

17. Abignano G, Aydin SZ, Castillo-Gallego C, et al. Virtual skin biopsy by optical coherence tomography: the first quantitative imaging biomarker for scleroderma. *Ann Rheum Dis*. 2013;72(11):1845-1851.

18. Su P, Cao T, Tang MB, Tey HL. In vivo high-definition optical coherence tomography: a bedside diagnostic aid for morphea. *JAMA Dermatol*. 2015;151(2):234-235.

19. Qiu X, Gong L, Lu Y, Jin H, Robitaille M. The diagnostic significance of Fourier-domain optical coherence tomography in Sjogren syndrome, aqueous tear deficiency and lipid tear deficiency patients. *Acta Ophthalmol*. 2012;90(5):e359-366.

20. Grulkowski I, Nowak JK, Karnowski K, et al. Quantitative assessment of oral mucosa and labial minor salivary glands in patients with Sjogren's syndrome using swept source OCT. *Biomed Opt Express*. 2013;5(1):259-274.

21. Ozawa N, Sumi Y, Shimozato K, Chong C, Kurabayashi T. In vivo imaging of human labial glands using advanced optical coherence tomography. *Oral Surg Oral Med Oral Pathol Oral Radiol Endod*. 2009;108(3):425-429.

22. Tian L, Qu JH, Zhang XY, Sun XG. Repeatability and Reproducibility of Noninvasive Keratograph 5M Measurements in Patients with Dry Eye Disease. *J Ophthalmol*. 2016;2016:8013621.

23. Paczesny S, Hakim FT, Pidala J, et al. National Institutes of Health Consensus Development Project on Criteria for Clinical Trials in Chronic Graft-versus-Host Disease: III. The 2014 Biomarker Working Group Report. *Biol Blood Marrow Transplant*. 2015;21(5):780-792.
