# Supplementary material for: Study protocol: Close Assessment and Testing for Chronic Graft-vs.-Host disease (CATCH)
Source: PLoS One. 2024 May 16;19(5):e0298026. doi: 10.1371/journal.pone.0298026 (PMC11098321; doi:10.1371/journal.pone.0298026)
Supplement: S1 Appendix — (DOCX) [file pone.0298026.s003.docx]

**Appendix. Participating sites from the Chronic GVHD Consortium**

Fred Hutchinson Cancer Center

Cleveland Clinic

H. Lee Moffitt Cancer Center and Research Institute

Roswell Park Cancer Institute

National Cancer Institute, Center for Cancer Research

University of Florida

Vanderbilt University
