# Supplementary material for: Study protocol: Close Assessment and Testing for Chronic Graft-vs.-Host disease (CATCH)
Source: PLoS One. 2024 May 16;19(5):e0298026. doi: 10.1371/journal.pone.0298026 (PMC11098321; doi:10.1371/journal.pone.0298026)
Supplement: Peer review history

# Editor report: Decision Letter 0

**DOI:** 10.1371/journal.pone.0298026.r001

**Authors:** Miquel Vall-llosera Camps

17 Jan 2024

Study Protocol: Close Assessment and Testing for Chronic Graft-vs.-Host Disease (CATCH)

PONE-D-23-39767

Dear Dr. Lee,

We’re pleased to inform you that your manuscript has been judged scientifically suitable for publication and will be formally accepted for publication once it meets all outstanding technical requirements.

Within one week, you’ll receive an e-mail detailing the required amendments. When these have been addressed, you’ll receive a formal acceptance letter and your manuscript will be scheduled for publication.

An invoice for payment will follow shortly after the formal acceptance. To ensure an efficient process, please log into Editorial Manager at http://www.editorialmanager.com/pone/, click the 'Update My Information' link at the top of the page, and double check that your user information is up-to-date. If you have any billing related questions, please contact our Author Billing department directly at authorbilling@plos.org.

If your institution or institutions have a press office, please notify them about your upcoming paper to help maximize its impact. If they’ll be preparing press materials, please inform our press team as soon as possible -- no later than 48 hours after receiving the formal acceptance. Your manuscript will remain under strict press embargo until 2 pm Eastern Time on the date of publication. For more information, please contact onepress@plos.org.

Kind regards,

Miquel Vall-llosera Camps, Ph.D.

Staff Editor

PLOS ONE

Journal requirements:

 

1. When submitting your revision, we need you to address these additional requirements.

 

Please ensure that your manuscript meets PLOS ONE's style requirements, including those for file naming. The PLOS ONE style templates can be found at 

https://journals.plos.org/plosone/s/file?id=wjVg/PLOSOne_formatting_sample_main_body.pdf and 

https://journals.plos.org/plosone/s/file?id=ba62/PLOSOne_formatting_sample_title_authors_affiliations.pdf.

 

2. Thank you for stating the following in the Competing Interests section: 

[JP has been a consultant and advisory board member for Syndax, CTI Biopharma, Amgen, Regeneron, Incyte. He has received clinical trial support from Novartis, Amgen, Takeda, Janssen, Johnson and Johnson, Pharmacyclics, Abbvie, CTI Biopharma, and Bristol Myers Squibb.

SZP received research support from the Center for Cancer Research at the National Cancer Institute through the National Institutes of Health Intramural Research Program, which includes Clinical Research Development Agreements with Celgene, Actelion, Eli Lilly, Pharmacyclics and Kadmon Corporation.

NF Incyte Advisory Board

SJL has received consulting fees from Mallinckrodt, Equillium, Kadmon, Novartis and Incyte; research funding from Amgen, AstraZeneca, Incyte, Kadmon, Novartis, Pfizer, Sanofi, Syndax and Takeda, and drug supply from Janssen. She is on Incyte and Sanofi clinical trial steering committees.]. 

 

Please confirm that this does not alter your adherence to all PLOS ONE policies on sharing data and materials, by including the following statement: ""This does not alter our adherence to  PLOS ONE policies on sharing data and materials.” (as detailed online in our guide for authors http://journals.plos.org/plosone/s/competing-interests). If there are restrictions on sharing of data and/or materials, please state these. Please note that we cannot proceed with consideration of your article until this information has been declared. 

 

Please respond by return email with your amended Competing Interests Statement and we will change the online submission form on your behalf.

 

3. We note that you have indicated that there are restrictions to data sharing for this study. PLOS only allows data to be available upon request if there are legal or ethical restrictions on sharing data publicly. For more information on unacceptable data access restrictions, please see http://journals.plos.org/plosone/s/data-availability#loc-unacceptable-data-access-restrictions. 

 

Before we proceed with your manuscript, please address the following prompts:

 

a) If there are ethical or legal restrictions on sharing a de-identified data set, please explain them in detail (e.g., data contain potentially identifying or sensitive patient information, data are owned by a third-party organization, etc.) and who has imposed them (e.g., a Research Ethics Committee or Institutional Review Board, etc.). Please also provide contact information for a data access committee, ethics committee, or other institutional body to which data requests may be sent.

 

b) If there are no restrictions, please upload the minimal anonymized data set necessary to replicate your study findings to a stable, public repository and provide us with the relevant URLs, DOIs, or accession numbers. For a list of recommended repositories, please see

https://journals.plos.org/plosone/s/recommended-repositories. You also have the option of uploading the data as Supporting Information files, but we would recommend depositing data directly to a data repository if possible.

 

We will update your Data Availability statement on your behalf to reflect the information you provide.

 

4.Please include your full ethics statement in the ‘Methods’ section of your manuscript file. In your statement, please include the full name of the IRB or ethics committee who approved or waived your study, as well as whether or not you obtained informed written or verbal consent. If consent was waived for your study, please include this information in your statement as well. 

 

5. Please include captions for your Supporting Information files at the end of your manuscript, and update any in-text citations to match accordingly. Please see our Supporting Information guidelines for more information: http://journals.plos.org/plosone/s/supporting-information. 

Additional Editor Comments:

Study Protocols that have been peer reviewed by an impartial, external funding body may be accepted without further peer review. Your paper has been assessed suitable for expedited handling.

# Editor report: Acceptance letter

**DOI:** 10.1371/journal.pone.0298026.r002

**Authors:** Miquel Vall-llosera Camps

2 May 2024

PONE-D-23-39767 

PLOS ONE

Dear Dr. Lee, 

I'm pleased to inform you that your manuscript has been deemed suitable for publication in PLOS ONE. Congratulations! Your manuscript is now being handed over to our production team.

At this stage, our production department will prepare your paper for publication. This includes ensuring the following:

* All references, tables, and figures are properly cited

* All relevant supporting information is included in the manuscript submission,

* There are no issues that prevent the paper from being properly typeset

If revisions are needed, the production department will contact you directly to resolve them. If no revisions are needed, you will receive an email when the publication date has been set. At this time, we do not offer pre-publication proofs to authors during production of the accepted work. Please keep in mind that we are working through a large volume of accepted articles, so please give us a few weeks to review your paper and let you know the next and final steps. 

Lastly, if your institution or institutions have a press office, please let them know about your upcoming paper now to help maximize its impact. If they'll be preparing press materials, please inform our press team within the next 48 hours. Your manuscript will remain under strict press embargo until 2 pm Eastern Time on the date of publication. For more information, please contact onepress@plos.org.

If we can help with anything else, please email us at customercare@plos.org.

Thank you for submitting your work to PLOS ONE and supporting open access. 

Kind regards, 

PLOS ONE Editorial Office Staff

on behalf of

Dr. Miquel Vall-llosera Camps 

Staff Editor

PLOS ONE
